# The metabolic response of marine copepods to environmental warming and ocean acidification in the absence of food

**DOI:** 10.1038/srep13690

**Published:** 2015-09-14

**Authors:** Daniel J. Mayor, Ulf Sommer, Kathryn B. Cook, Mark R. Viant

**Affiliations:** 1 Institute of Biological and Environmental Sciences, Oceanlab, University of Aberdeen, Main Street, Newburgh, Aberdeenshire AB41 6AA, UK; 2 NERC Biomolecular Analysis Facility – Metabolomics Node (NBAF-B), School of Biosciences, University of Birmingham, Birmingham, B15 2TT, UK; 3 Marine Scotland Science, Scottish Government, Marine Laboratory, 375 Victoria Road, Aberdeen AB11 9DB, UK; 4 Ocean Biogeochemistry and Ecosystems, National Oceanography Centre, Southampton, SO14 3ZH, UK

## Abstract

Marine copepods are central to the productivity and biogeochemistry of marine ecosystems. Nevertheless, the direct and indirect effects of climate change on their metabolic functioning remain poorly understood. Here, we use metabolomics, the unbiased study of multiple low molecular weight organic metabolites, to examine how the physiology of *Calanus* spp. is affected by end-of-century global warming and ocean acidification scenarios. We report that the physiological stresses associated with incubation without food over a 5-day period greatly exceed those caused directly by seawater temperature or pH perturbations. This highlights the need to contextualise the results of climate change experiments by comparison to other, naturally occurring stressors such as food deprivation, which is being exacerbated by global warming. Protein and lipid metabolism were up-regulated in the food-deprived animals, with a novel class of taurine-containing lipids and the essential polyunsaturated fatty acids (PUFAs), eicosapentaenoic acid and docosahexaenoic acid, changing significantly over the duration of our experiment. Copepods derive these PUFAs by ingesting diatoms and flagellated microplankton respectively. Climate-driven changes in the productivity, phenology and composition of microplankton communities, and hence the availability of these fatty acids, therefore have the potential to influence the ability of copepods to survive starvation and other environmental stressors.

Marine copepods of the genus *Calanus* dominate zooplankton biomass from the North Sea to the Arctic^1^. *Calanus* contributes significantly to a range of ecosystem services that benefit mankind. They provide a crucial trophic link between phytoplankton and juvenile fish, and hence are important for energy transfer and the production of commercially harvestable biomass^2^,^3^. They also contribute significantly to biogeochemical cycles, most notably by sustaining phytoplankton production in the upper ocean via ammonia excretion^4^ and by exporting carbon into the deep-sea by means of their large, densely packed faecal pellets^5^.

*Calanus* spp. are adapted to survive periods of food deprivation caused by the spatial heterogeneity of their microplankton prey and the strong seasonality of the high-latitude ecosystems that they inhabit. Indeed, one of their most striking adaptations is the seasonal acquisition of energy-rich lipids in a central body sac^6^,^7^, which can constitute >50% of their dry weight. Lipids serve as a metabolic reserve during diapause, the overwintering process that involves these animals descending into the deep ocean and entering a period of torpor that may last for ≥6 months. Lipids are also used, along with body proteins, to fuel mass spawning events that occur in advance of the spring bloom^8^, thereby ensuring that the next generation coincides with good feeding conditions.

The arrival of spring temperatures in the waters of the northern hemisphere advanced at a rate of 2 days per decade between 1960 and 2009^9^. This progressive warming has enhanced the development rates of copepods like *Calanus* spp. but not their diatom prey, resulting in a trophic mismatch that is set to increase as warming continues^10^,^11^. Warming has further, negative impacts upon marine primary production, and hence copepod feeding conditions, through enhanced thermal stratification and the consequential reduction of nutrient fluxes into the photic zone^12^,^13^.

Recent decades have seen considerable changes to the biogeographical ranges of *Calanus* spp. throughout the North Atlantic^14^ and a 70% decline in their overall abundance in the North Sea since the 1960’s^15^. Both of these phenomena have been attributed to the North Atlantic Oscillation and increased sea surface temperatures throughout the region^16^. Ocean acidification, caused by the increased uptake of anthropogenic CO_2_ in seawater, has occurred in concert with warming during this observational period. Whether or not this phenomenon has contributed to the decline in *Calanus* spp. remains unknown.

Animals are adapted to survive within a ‘thermal window’, with temperature shifts away from optimum resulting in reduced performance and ultimately death unless specific adaptations are present, migration occurs or the rate of change is slow enough to permit adaptation^17^. Exposure to elevated CO_2_ concentrations may place an additional constraint on an organism’s thermal window and hence reduce their performance^17^. Acid-base equilibria in the body fluids of crustaceans are maintained through a variety of mechanisms, many of which are metabolically costly^18^. A recent study demonstrated that feeding rates of the marine copepod, *Centropages tenuiremis*, exposed to 1000 ppm CO_2_, were elevated relative to the controls, apparently compensating for the observed CO_2_-driven increase in respiration rates^19^. The extent to which compensatory feeding occurs in the natural environment remains unknown, and it can only occur where feeding conditions permit. External stressors such as ocean acidification are therefore expected to reduce the quantities of resources that would otherwise be available for growth and hence affect long-term reproductive output. Indeed, it is quite possible that the observed decline in *Calanus* spp. throughout the North Sea reflects direct, chronic effects of a warmer and more acidic ocean at the metabolic level that are exacerbated by reduced access to food, itself an indirect effect of environmental warming. Future warming and ocean acidification scenarios are both reported to affect the development and reproductive potential of *Calanus* spp.^20^,^21^,^22^. We are unaware of any studies that have simultaneously examined how warming and acidification affect metabolic processes and hence the performance of these animals.

Environmental metabolomics is the study of how the metabolic profile of an organism responds to changes in the external environment. This field of research has demonstrated that an organism’s metabolome, the suite of low molecular weight organic metabolites within their tissues and biofluids, changes in response to intrinsic processes such as growth and reproduction and also extrinsic factors such as temperature, food availability and contaminant exposure^23^. A major benefit of the metabolomics approach is that it studies multiple metabolites equally and is therefore not biased or constrained by our contemporary understanding of physiology. Metabolomic techniques have recently been applied to better understand how the physiology of *Calanus* spp. responds to external stressors^24^,^25^. The present study used a factorial experimental design in combination with direct infusion mass spectrometry (DIMS) based metabolomics^26^ to investigate how exposure to predicted end-of-century atmospheric conditions, +2 °C and 1000 μatm *p*CO_2_, affected the metabolic profile of a mixture of *C. finmarchicus* and *C. helgolandicus*. We hypothesized that the effects of food deprivation on the metabolome of *Calanus* spp. would be exacerbated by the effects of warming and seawater acidification. More specifically, we expected starvation-induced turnover of lipids and proteins to be elevated in our climate change treatments owing to the additional metabolic demands that these stressors impose.

## Results

Two hundred individual *Calanus* spp. CVs were incubated for a period of 5 days in the absence of food, with replicate (n = 10) groups of 5 animals exposed to ambient or 1000 μatm *p*CO_2_ at either 8 °C or 10 °C (Table 1). A total of 22 animals died during the experiment and a further 17 were lost due to the rapid handling of samples immediately prior to flash freezing. Mortality and handling losses were not attributable to differences in temperature (ANOVA, 1df, p = 0.685 and 0.106 respectively) or CO_2_ concentration (ANOVA, 1df, p = 0.217 and 0.241 respectively).

**Figure 1:**
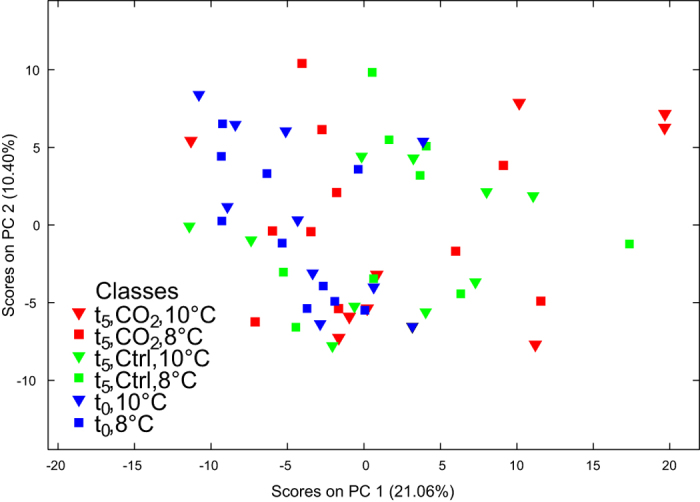
The response of polar metabolites extracted from *Calanus* spp. Principal component analysis of the FT-ICR mass spectra of the polar metabolites in experimental animals following treatments at ambient (Ctrl) and elevated *p*CO_2_ (CO2) levels at 8 °C and 10 °C (8C and 10C) over five days (t_0_ = pre-experimental animals; t_5_ = post-experimental animals). Results for the test for significant separations along the PCs can be found in Table S1.

**Figure 2:**
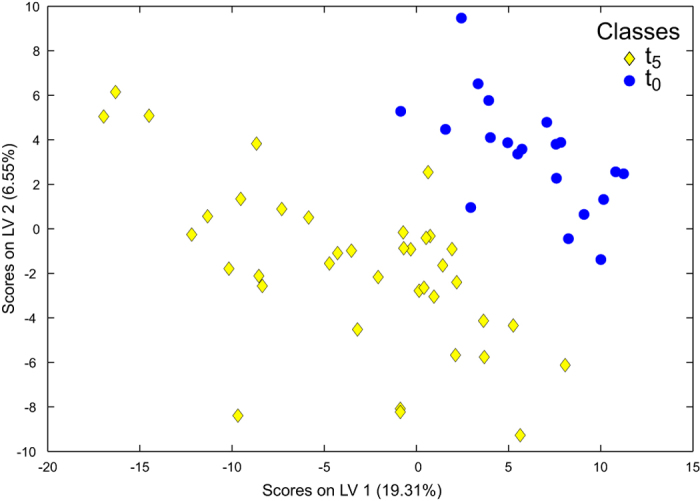
Discrimination of the polar metabolites extracted from *Calanus* spp. by experimental time point. Partial least squares discriminant analysis of the FT-ICR mass spectra highlighting the metabolic differences between the pre-experimental (t_0_) and post-experimental (t_5_) animals (classification error rate 3.3%, p < 0.001, Table S2). Incubations were conducted in the absence of food.

Principal components analysis (PCA) of the polar metabolite data, which contained an intensity data matrix and peaklist of 2487 signals, is shown in Fig. 1. The first principal component described a significant amount of the variance between treatment groups (21.06%, p = 0.018; Supplementary Table S1). The dominant metabolic effect was revealed to be the slight separation of samples from the pre-experimental animals (t_0_), which were negatively correlated with PC1 (Fig. 1). Specifically, metabolic profiles of t_0_ animals acclimated to 8 °C were different to those of post-experimental animals (t_5_) exposed to 1000 μatm *p*CO_2_ at 10 °C (Supplementary Table S1). Differences between the four individual treatment groups at the end of the experiment (t_5_) were not significant (Supplementary Table S1). The trend identified in the PCA was explored by re-grouping the samples accordingly to experimental day (t_0_ vs. t_5_) using partial least squares discriminant analysis (PLS-DA), a form of supervised multivariate analysis. Differences between the metabolic profiles at t_0_ and t_5_ were highly significantly (p < 0.001; Fig. 2; Table 2) and were attributable to 94 signals in the FT-ICR mass spectra (Supplementary Tables S2 & S3). Univariate comparisons (t-tests) between signals from the t_0_ and t_5_ samples indicated that 316 signals were significantly different (defined as q < 0.05; Supplementary Table S3), of which 48 were also highlighted as important by the PLS-DA model. The data were further interrogated by removing the t_0_ samples, thereby allowing the metabolic profiles associated with the remaining four treatment groups at t_5_ to be more thoroughly compared. All of the resulting PLS-DA models lacked predictive power, reflected by their high classification error rates and non-significant separation of groups (Table 2). These results indicated that there were no overall metabolic differences between the four treatment groups, a finding that was supported by further univariate statistical analyses which failed to discern significant differences between any of the t_5_ experimental treatment groups (q ≥ 0.423 in all cases).

**Figure 3:**
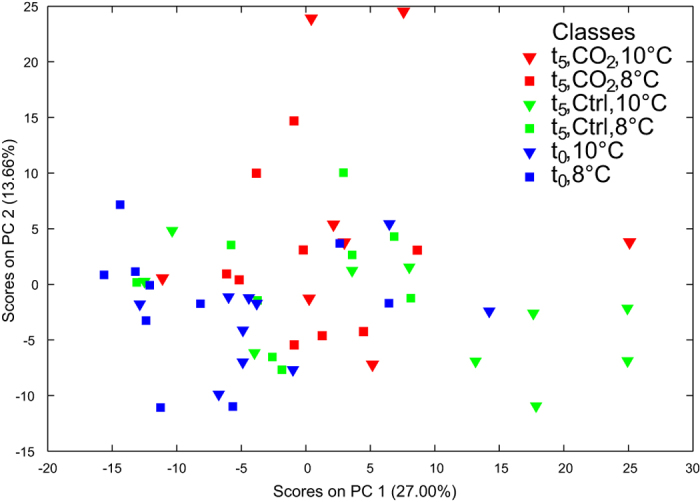
The response of nonpolar metabolites extracted from *Calanus* spp. Principal component analysis of the FT-ICR mass spectra of the nonpolar metabolites in experimental animals following treatments at ambient (Ctrl) and elevated *p*CO_2_ (CO2) levels at 8 °C and 10 °C (8C and 10C) over five days (t_0_ = pre-experimental animals; t_5_ = post-experimental animals). Results for the test for significant separations along the PCs can be found in Table S4.

**Figure 4:**
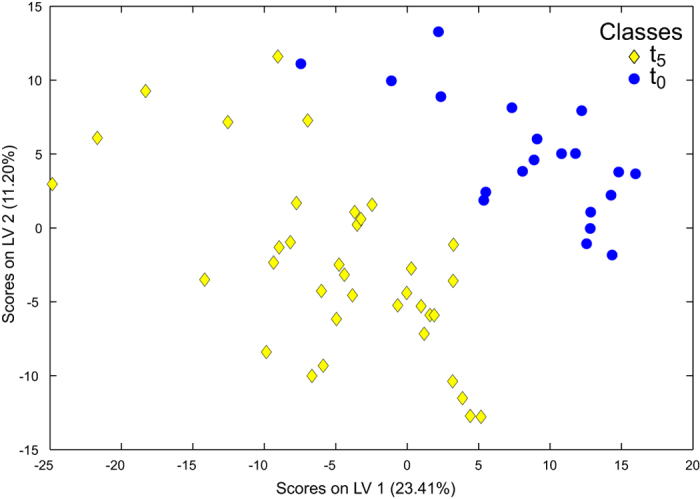
Discrimination of the nonpolar metabolites extracted from *Calanus* spp. by experimental time point. Partial least squares discriminant analysis of the FT-ICR mass spectra highlighting the metabolic differences between the pre-experimental (t_0_) and post-experimental (t_5_) animals (classification error rate 4.0%, p < 0.001, Table S5). Incubations were conducted in the absence of food.

Principal components analysis of the non-polar metabolite data, which contained an intensity data matrix and peaklist of 1771 signals, is shown in Fig. 3. The first three principal components all explained a significant amount of the variance between treatment groups (p ≤ 0.032 in all cases; Supplementary Table S4), with the major separation again being driven by differences between t_0_ and t_5_. Nonpolar metabolite profiles from the t_0_ animals acclimated to 8 °C were different to those from t_5_ animals exposed to ambient and 1000 μatm *p*CO_2_ at 10 °C (Supplementary Table S4). The t_5_ animals exposed to 1000 μatm *p*CO_2_ at 10 °C could also be statistically distinguished from t_0_ and t_5_ animals from the ambient CO_2_ treatments that were incubated at 10 °C (Supplementary Table S4). Other differences between the four individual treatment groups at the end of the experiment (t_5_) were not significant (Supplementary Table S4). The PLS-DA model derived from the analysis of the data that was re-grouped by experimental day (t_0_ vs. t_5_) again revealed highly significant differences between these time points (p < 0.001; Fig. 4; Table 2). This effect was attributable to 101 signals in the FT-ICR mass spectra (Supplementary Tables S5 & S6). Univariate analysis of the data grouped by experimental day revealed that 261 of the signals at t_5_ were significantly different to those at t_0_ following false discovery rate (FDR)-correction (defined as q < 0.05; Supplementary Table S6). Statistical interrogation, using PLS-DA, of the nonpolar data with the t_0_ samples removed found no differences between the CO_2_ treatments at either 8 °C or 10 °C (Table 2). However, when the t_5_ data were re-grouped by CO_2_ treatment alone, i.e. the temperature treatments were pooled, the metabolic profiles of animals exposed to 1000 μatm *p*CO_2_ were marginally different to those at ambient CO_2_ (p = 0.041; Supplementary Figure S1), albeit with a high 32% classification error rate. While this PLS-DA model suggested that 61 signals in the mass spectra were important, FDR-corrected univariate comparisons only discerned one significantly different signal (q = 0.023, next lowest q = 0.245). A marginally significant effect of temperature was apparent when data from the two t_5_ CO_2_ treatments were pooled and subject to PLS-DA (p = 0.051; Supplementary Figure S2). The classification error rate for this model was 35% (Table 2), and the PLS-DA model included only 40 important signals; FDR-corrected univariate comparisons could not discern any significantly different signals (q ≥ 0.304 in all cases).

**Figure 5:**
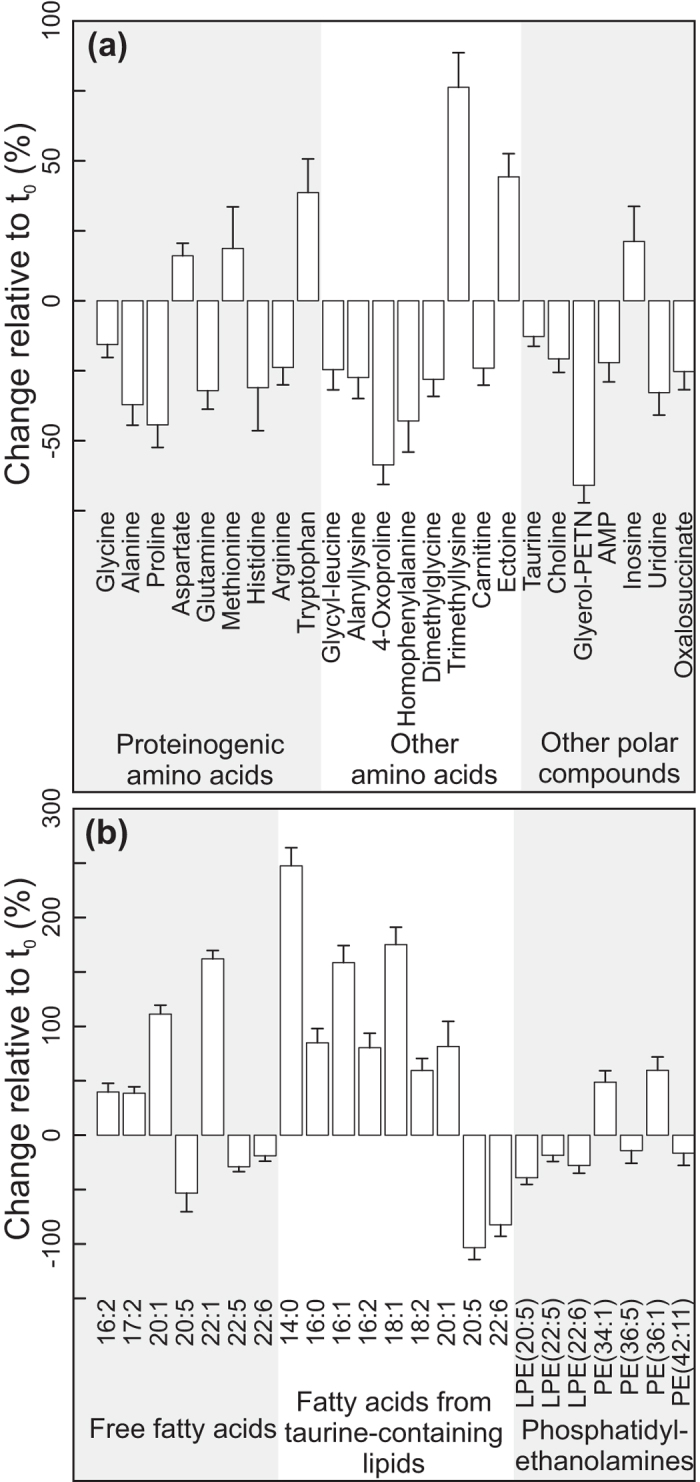
Relative changes of key metabolites in *Calanus* spp. between pre- and post-experimental time points. Levels of selected (**a**) polar and (**b**) nonpolar metabolites after a 5-day period of incubation in the absence of food (t_5_), relative to individuals collected at the outset of the experiment (t_0_). Values are expressed as percentages (average ± standard error). All of these metabolites contributed significantly (q < 0.05, or forward selected in PLS-DA models) to discriminating between the time points.

Of the 2487 signals (*m/z* values) measured in the polar *Calanus* spp. extracts (Supplementary Table S3), 466 matched entries in the KEGG database, 378 could not be assigned a molecular formula within a 1 ppm search criteria, and the remaining signals were assigned one or more molecular formula(e) but were not present in KEGG (of these, 422 each matched to only one empirical formula). The majority of the putatively annotated polar metabolites that contributed significantly to distinguishing the t_0_ and t_5_ animals decreased in intensity over the duration of the experiment (Fig. 5a). These metabolites included the two most common free amino acids glycine and arginine (Supplementary Figure S3) and the nitrogen-rich amino acids glutamine, arginine and histidine. Aspartate, methionine and tryptophan were the only proteinogenic amino acids that increased in intensity from t_0_ to t_5_. Most of the amino acid derivatives, including the osmolyte taurine, and the other identified polar compounds also occurred in lower abundance in the t_5_ animals. Oxaloacetate, the only identified metabolite associated with the citric acid cycle that changed significantly, also decreased in intensity throughout the 5-day experiment. A group of ions from the polar extracts could be identified as Li^+^ adducts, as indicated by their ^6^Li isotopes (ca. 8% intensity at −1.0009 Da) (Supplementary Figure S4). While a number of these signals changed significantly between t_0_ and t_5_, they appear to be adducts of compounds which otherwise are not of interest.

Of the 1771 signals (*m/*z values) measured in the nonpolar extracts (Supplementary Table S6), 815 matched entries in the Lipid Maps database within the 1.5 ppm search criteria. Some of these, however, related to classes of compounds rather than individual lipids. An additional search for peaks up to *m/z* 600, which included 913 of the 1771 signals, yielded 343 signal matches in the KEGG database and provided single, exact molecular formulae for 38 of these; 45 signals could not be assigned a formula (Supplementary Table S6). The annotation of an important group of nonpolar compounds that contributed significantly to distinguishing the t_0_ and t_5_ animals required further mass spectrometric analyses (Supplementary Table S7). These compounds were characterized as a family of novel taurine lipids. Our mass spectrometry data show losses of (different sized) fatty acids from a fixed backbone of taurine attached to a unit of 306 Da (C_20_H_34_O_2_) (Supplementary Figures S5-S8). Further nonpolar compounds that differentiated the t_0_ and t_5_ animals were distinguished by MS/MS fragmentation from isobaric analytes as being phosphatidylethanolamine, phosphatidylcholine and a free fatty acid (Supplementary Figures S9-S11). The nonpolar compounds that contributed most significantly to discriminating between the t_0_ animals from those incubated for 5 days (t_5_) belonged to three classes; free fatty acids, fatty acids from taurine-containing lipids and (lyso)phosphatidylethanolamines (Fig. 5b). The patterns observed across these groups were generally consistent; non-essential, typically saturated or monounsaturated compounds increased over the duration of the experiment, whereas the polyunsaturated moieties, including eicosapentaenoic acid (EPA), docosahexaenoic acid (DHA) (annotated as 20:5 and 22:6 respectively) and their related residues decreased in all three classes. The simple phospholipids sphingosine 1-phosphate and sphingosyl-phosphocholine did not change over the experimental duration (Supplementary Table S6).

## Discussion

Our data suggest that the metabolic performance of sub-adult *Calanus* spp. remains unaffected by short-term exposure to the warmer and more acidic conditions that these animals are expected to face by the end of 2100; all univariate comparisons attempting to ascribe changes in the polar- and nonpolar datasets to the effects of temperature and/or *p*CO_2_ were non-significant. The lack of clear temperature effects may, in part, reflect that the animals were acclimated to their exposure temperatures for 72 hrs prior to incubation. This argument does not explain our inability to associate changes in any metabolites with the short-term, shock exposure to what amounts to almost a tripling of the ambient seawater *p*CO_2_. The apparently benign, direct effects of temperature and elevated CO_2_ concentration on the metabolome of *Calanus* spp. could reflect the variable histories of our wild-caught experimental animals^20^,^24^,^27^, or that we incubated a mixture of *Calanus helgolandicus* and *Calanus finmarchicus*. Recent work has demonstrated that the metabolomes of wild-caught *C. finmarchicus*, *C. glacialis* and *C. hyperboreus* can be statistically discriminated, albeit with the differences being predominantly driven by *C. hyperboreus*, the much larger and more ecologically dissimilar species see Tables 2 & 3 in ref. 25. We cannot, therefore, exclude the possibility that potential differences between the metabolomic profiles of *C. finmarchicus* and *C. helgolandicus* confounded our ability to statistically discern the effects of temperature and/or CO_2_ concentration. These organisms apparently occupy different thermal niches^28^, presumably reflecting as-yet not understood physiological differences that may have introduced a degree of noise into our metabolic profiles. However, *C. finmarchicus* and *C. helgolandicus* co-exist and grow to an identical size within regions such as the North Sea, where temperatures are the same as in our experiment^29^. The strict allometric scaling of copepod metabolism^30^ and other strong biochemical and ecological similarities between these sympatric species^28^ collectively suggest that they share very similar metabolic traits. It therefore seems highly unlikely that their metabolic responses to temperature and/or CO_2_ concentration would be sufficiently dissimilar to mask the manifestation of any such responses in our experimental data. Indeed, we contend that our inability to discern treatment effects cannot be attributed to working with a mixture of *C. finmarchicus* and *C. helgolandicus* and explore alternative explanations in the following text.

We deliberately conducted our experiment in the absence of food to simultaneously examine the effects of food deprivation whilst avoiding the confounding effects of treatment-induced differences in feeding^19^,^31^ and their impact on the metabolomes of our experimental animals^32^. Our experimental design, and the absence of food in particular, could have masked more subtle effects of temperature and pH. However, we suggest that the metabolism of sub-adult *Calanus* spp. is unlikely to be significantly impaired by end of century CO_2_-incuded acidification and changes in temperature, at least in the short term. This understanding is in contrast to what may be anticipated on the basis of the known physiological response of larger, calcifying crustaceans to decreased pH^18^ and the observed response of the coastal copepod, *Centropages tenuiremis*^19^. Nevertheless, several lines of evidence support our interpretation: a) *C. finmarchicus* and *C. helgolandicus* display pronounced diel vertical migration, a trait that enables them to evade visual predators and the metabolic expense of inhabiting the warm, surface waters during the day. This behaviour also exposes them to a range of *p*CO_2_ conditions, which can show considerable vertical and horizontal variability^33^. Animals that regularly face exposure to variable seawater temperature and *p*CO_2_ are hypothesized to be more resilient to the effects of ocean acidification and warming owing to the necessity for physiological adaptations^34^,^35^. The observation that vertically migrating copepods, including a variety of *Calanus* spp., appear to be robust to end of century climate scenarios^33^ lends weight to this understanding; b) Previous studies have demonstrated that rates of respiration, biomass turnover and lipid accumulation in *C. finmarchicus* exposed to CO_2_-acidified seawater at pH 7.3–~6.9 do not differ to their values in the corresponding control treatments^22^,^36^,^37^. Experiments in which sub-adults and females of the congeners, *Calanus glacialis* and *Calanus hyperboreus,* were exposed to 3000 μatm *p*CO_2_ (pH ~7.3) also failed to discern treatment effects on the rates of respiration and biomass turnover^38^; c) Gonad development and egg production in *Calanus* spp. are energetically demanding^39^, yet these processes are reported to be unaffected by exposure to seawater acidified with CO_2_ between pH ~7.3–~6.9^21^,^37^,^38^.

Our interpretation does not necessarily imply that *Calanus* spp. populations will remain unaffected by the direct effects of future environmental change. There is growing consensus that the eggs and younger developmental stages of invertebrates are disproportionally sensitive to ocean acidification^40^. Indeed, a range of studies demonstrate that egg hatching success and the subsequent rates of survival and development of the nauplii and copepodites in *Calanus* spp. are reduced by exposure to acidified seawater^21^,^22^,^33^,^37^,^38^. Multi-generational studies with animals of known exposure history are required to better understand the long-term impacts of future environmental scenarios^41^. However, data derived from such experiments require careful interpretation as stable laboratory conditions will differ considerably to the bio-physiochemical heterogeneity experienced by wild-type specimens in the natural environment^6^.

*Calanus* spp. face periods when the ingested daily ration fails to meet their metabolic demands^8^,^42^. Global warming is thought to indirectly exacerbate this effect by causing a trophic mismatch between copepods and their diatom prey^10^,^11^ and by reducing marine primary production^12^,^13^. Our data demonstrate that 5 days of incubation in the absence of food is sufficient to elicit significant changes in both the polar- and non-polar metabolome of *Calanus* spp., with clear impacts upon various aspects of lipid and protein metabolism. The most substantial changes observed in the metabolome of our experimental animals relate to what we term taurine-containing lipids (Fig. 5b). This class of compounds may ultimately be considered as a metabolic signature of starvation in *Calanus* spp., although further work is required to verify this suggestion. Our preliminary mass spectrometry-based characterization of these taurine-containing lipids suggests that they are comprised of a variable fatty acid residue attached to an invariable part consisting of taurine and a 20-carbon unit component (C_20_H_34_O_2_) (Supplementary Figures S5-S7). They may be similar to the taurolipid structures found in the ciliated protozoan, *Tetrahymena thermophila*^43^ (Supplementary Figure S8a). We speculate that the taurine-containing lipids in *Calanus* spp. play an active role in the transportation of metabolic substrates from their storage location to the point of catabolism^44^ (Supplementary Text S1). A contemporaneous study has described a class of similar, if not identical compounds as ‘copepodamides’, which are reported to stimulate the production of toxins in a bloom-forming dinoflagellate^45^ (Supplementary Figure S8b). Evidently these compounds warrant further investigation.

The levels of many of the unsaturated and monounsaturated free fatty acids and those from the taurine-containing lipids increased over the duration of our experiment (Fig. 5b). The absence of food in our incubations necessitates that these observations indicate the catabolism of previously sequestered lipid reserves, and possibly also the elongation and/or desaturation of their constituent fatty acids as they are readied for catabolism. Major increases of the free fatty acids 20:1(ω-9) and 22:1(ω-11) (denoted as 20:1 and 22:1 respectively herein) suggest the catabolism of wax esters as these compounds are biosynthesized by calanoid copepods and predominantly stored in this moiety^46^. Protein and amino acid metabolism in copepods has received considerably less attention than lipid metabolism and it is therefore not possible to ascribe all of the observed changes in the amino acids or their derivatives to particular pathways. Nevertheless, increases in the free amino acids aspartate, methionine and tryptophan in the post-experimental animals indicate the catabolism of proteins, a process that is known to contribute to the metabolic demands of *Calanus* spp. when food is scarce^8^,^47^. The fact that the most abundant and nitrogen-rich amino acids, e.g. histidine (C_6_H_9_N_3_O_2_) and arginine (C_6_H_14_N_4_O_2_), declined suggests that these are preferred sources for catabolic purposes, while the decline of others might be derived from their direct use, or from the decline of precursor compounds (*e.g.*, proline). The substantial increase in trimethyllysine, which is produced via protein hydrolysis^48^, is interesting, not least because this amino acid derivative is also reported to increase significantly during starvation in the freshwater amphipod *Diporea* spp.^49^. Trimethyllysine is an important precursor for carnitine, which is involved in the regulation of fatty acid transport across the mitochondrial membrane prior to β-oxidation^50^. However, the observed decline in carnitine suggests that this was not the primary fate for this compound in our experimental animals. The up-regulation of trimethyllysine more likely relates to its role as an active co-enzyme in fatty acid oxidation: The ω-3 polyunsaturated fatty acids (PUFAs) eicosapentaenoic acid (EPA, 20:5(ω-3)) and docosahexaenoic acid (DHA, 22:6(ω-3)) (denoted 20:5 and 22:6 respectively herein) and their taurine- and phosphatidylethanolamine residues were all lower in the post-experimental animals, along with many amino acids and other polar compounds (Fig. 5b). These observations are consistent with the understanding that lipid catabolism plays a significant role in *C. finmarchicus* and *C. helgolandicus* during periods of food deprivation^6^,^8^,^47^, and more specifically, the polyunsaturated moieties^27^,^51^.

Starvation-induced turnover of ω-3 PUFAs has been observed in other crustaceans including branchiopods^52^,^53^, amphipods^49^,^54^ and other copepods^55^. Fish, including capelin^56^ and Japanese horse mackerel^57^ also catabolise significant quantities of EPA and DHA in the absence of food, suggesting that these compounds play a central role in the adaptations of aquatic organisms to food shortages. Catabolising PUFAs as a strategy to survive short-term starvation seems counterintuitive; these compounds yield less energy than their equivalent mono- or unsaturated moiety. Furthermore, EPA and DHA are essential fatty acids for both marine zooplankton^58^ and fish^59^. Reliance upon EPA and DHA as metabolic substrates during stressful situations may simply reflect the abundance of these compounds in the marine environment. However, the apparently selective catabolism of EPA and DHA by a range of different organisms suggests otherwise and there is growing appreciation that marine organisms carefully control the composition of their lipids at all times to ensure that ecological fitness is maximised^60^,^61^. EPA is a necessary precursor to a group of bioactive, hormone-like compounds called eicosanoids that are associated with a range of physiological processes, including stress responses, and their production is determined, in part, by competitive interactions with other PUFAs^59^. The unique biophysical properties of EPA have implicated this compound as a key agent through which *C. finmarchicus* may control the initiation and termination of diapause^51^. EPA is also thought to influence the depth at which the analogous calanoid copepod in the southern hemisphere, *Calanoides acutus*, attains neutral buoyancy in the deep sea during diapause^62^,^63^. DHA appears to be central in how *C. acutus* adapt their cellular membranes to maintain functionality across the considerable temperature- and pressure gradients they encounter when entering and exiting diapause^64^.

Significant biological demands and hence rapid turnover of EPA and DHA^27^ suggest that their role in limiting the growth of *Calanus* spp.^65^ and potentially other organisms may have previously been underestimated. Perhaps more importantly, these observations have major implications for how such animals will fare in the future. Warming-induced reductions in marine productivity^12^,^13^ and plankton phenology^10^,^11^ will both decrease the absolute quantities of food available to herbivorous zooplankton. These changes have additional consequences for the availability of PUFAs such as EPA and DHA, which are ultimately derived from diatoms and flagellated microplankton respectively. Equally, climate-driven changes in the composition of pelagic microplankton communities, such as the observed reduction in the relative abundance of dinoflagellates in the northeast Atlantic and North Sea^66^, also directly affect the supply of PUFAs and other essential biochemical compounds to marine food chains. The central roles of EPA and DHA in the physiology of important organisms such as *Calanus* spp. suggest that any changes in their supply will affect the ecological fitness of these animals that will likely have a negative impact at the population scale. Indeed, the importance of these compounds for fish has led to the suggestion that their dwindling availability in the plankton caused the observed regime shifts across the boreal continental shelves of the Pacific and Atlantic oceans, where lipid-rich pelagic fish species have been replaced by lipid-poor demersal species^67^. Could the dramatic decline of *Calanus* spp. in the North Sea over the past 50 years^15^ reflect a reduction in the supply of EPA and DHA, potentially an indirect consequence of climate change, rather than because of any direct impacts of warming and/or acidification on their physiology? We cannot mechanistically explain the apparent significance of EPA and DHA in our data, and are unaware of any other literature that has elucidated their apparently counterintuitive roles in the starvation response of other organisms. Nevertheless, a wealth of evidence suggests that they are intricately linked to the wider roles of EPA and DHA in the physiological ecology of *Calanus* spp., and further study is required to investigate the specific physiological mechanisms with which these compounds interact. We advocate the integrated use of contemporary analytical techniques, including metabolomics, transcriptomics and proteomics, and suggest that this will yield significant advances in our understanding of the physiological ecology of *Calanus* spp. and their response to environmental change.

## Methods

Our fully factorial experiment compared the metabolic profiles of 10 replicate groups of 5 wild-caught *Calanus* spp. stage 5 copepodites (so-called ‘CVs’) acclimated to 8 °C and 10 °C in 0.2 μm filtered seawater (FSW) to those of replicate groups of CVs incubated in FSW at 8 °C or 10 °C that had been equilibrated to ambient air, containing ~380 μatm pCO_2_ (ambient hereafter), or air enriched to 1000 μatm pCO_2_, for a period of 5 days in the absence of food (6 treatment regimes, n = 10 each, total n = 60; see Table 1). CO_2_-enriched air was commercially produced to order (BOC Special Gases, UK) (see ref. 20 for further details). Experimental animals were collected off Stonehaven, NE Scotland (56°57.80N, 02°06.20W) on 23 May 2011 using a 1 m ring net fitted with a 250 μm mesh net and non-filtering cod-end. The percentage abundance of *C. helgolandicus*:*C. finmarchicus* was 55:45 at the time of sampling. Zooplankton were gently concentrated onto a 45 μm mesh and 60 replicate groups of 5 *Calanus* spp. CVs were carefully picked into FSW; 30 of these replicates were acclimated to each of the two temperature regimes in ambient FSW for 72 hrs prior to experimentation. We chose to work with CVs to ensure that eggs were not available as a potential food source during our experiment. No attempts were made to distinguish between live *C. helgolandicus* and *C. finmarchicus* (*Calanus* spp. hereafter) because it is not possible to reliably achieve this without inflicting severe stress and/or killing the animals. At the outset of the experiment (t_0_), 10 replicate groups of 5 animals from the two temperatures were individually concentrated onto a 45 μm mesh, rapidly transferred into Precellys homogenisation tubes (Stretton Scientific, UK) and flash frozen in liquid nitrogen. The experimental setup was conceptually similar to that described previously^37^. Experimental animals were transferred into 500 ml egg production chambers containing FSW at the prescribed temperature and CO_2_ level, topped up with the appropriate FSW (ambient or 1000 μatm pCO_2_) and sealed with an air-tight lid to avoid CO_2_ out-gassing. These were incubated for 24 hrs and subsequently transferred into fresh FSW at the same temperature and CO_2_ level after each 24 hr incubation period for a total of 5 days. The experiment was conducted in the absence of food to simultaneously examine the effects of food deprivation. This also avoided any confounding effects of feeding differences between the treatments. At the end of the experiment (t_5_) animals were collected and flash frozen in liquid nitrogen as described above. All samples were stored frozen at −80 °C until analysis. The pH of the incubation water was measured at the start and end of each 24-hr period using a Hanna bench top meter (H1859) following calibration using reference standards at pH 4, 7 and 10 (Sigma, UK). Average pH values of the ambient and CO_2_-acidified experimental seawater were 8.09 ± 0.03 (sd) and 7.76 ± 0.08, respectively; values did not differ between the temperature treatments (F = 0.616, df = 1, p = 0.4337) or between the start and end of each 24 hr incubation period (F = 0.100, df = 1, p = 0.7522). Experimental treatments and seawater chemistry are summarised in Table 1.

The mass spectrometry based metabolomics workflow used here from extraction to statistical analysis has been summarised recently^68^. All mentioned in-house scripts are available on request. In brief, 100 μl methanol (HPLC grade) and 40 μl water (HPLC grade) per copepod were added to each sample prior to homogenization using a Precellys-24 ceramic bead-based homogenizer (Stretton Scientific Ltd., UK). Thereafter, 50 μl water (HPLC grade) and 100 μl chloroform (pesticide analysis grade) per copepod were added to each sample, which was vortexed and centrifuged (1800 *rcf*, 10 min, 4 °C), yielding an upper (polar) and lower (nonpolar) fraction for each sample. Each polar fraction was collected into a 1.5 ml Eppendorf tube, and the nonpolar fraction into a 1.8 ml glass vial. Extraction blanks were also prepared using identical methods except that no biological material was added to the solvents. Two quality control (QC) samples were prepared by pooling small aliquots from the polar and nonpolar extracts, respectively. All polar samples were dried in a centrifugal concentrator (Thermo Savant, Holbrook, NY), the nonpolar samples under a stream of nitrogen gas. All samples were stored at −80 °C prior to analysis.

Direct infusion mass spectrometry (DIMS) data acquisition in the positive ion mode was performed on a Fourier transform ion cyclotron resonance (FT-ICR) mass spectrometer (LTQ FT Ultra, Thermo Fisher Scientific, Bremen, Germany) with a chip-based Triversa nanoelectrospray source (Advion Biosciences, Ithaca, NY). The polar extracts were each taken up in 100 μl of 0.25% formic acid in methanol/water (4:1 v/v). Samples were centrifuged (22000 *rcf*, 10 min, 4 °C) to remove any particulate matter. They were analysed in a controlled-randomized sequence different from the metabolite extraction sequence, with three technical replicates measured for each sample. QC samples were analysed at the beginning, the end, and equidistantly throughout the sequence. Data were acquired at nominal 1E5 resolution from *m/z* 70–590, in seven wide-SIM (selected ion monitoring) windows. Nonpolar (lipid) samples were taken up in the original volume of methanol/chloroform (3:1 v/v), containing 5 mM ammonium acetate. Data were acquired in increasing SIM windows of 100 Da to 200 Da width, from *m/z* 120 to 1200. The nonpolar QC sample was used in the same way as for the polar analyses. The nonpolar samples were also analysed in a controlled-randomized order and each was measured in three technical replicates. Raw mass spectral data were processed using the SIM-stitching algorithm, using an in-house Matlab script. The data matrices were normalized using the PQN algorithm. Missing values were imputed using the KNN algorithm via an in-house R script. The resulting data matrix was analysed using univariate statistics, described below. The same matrix was transformed using the generalised logarithm to stabilise the technical variance across the measured peaks prior to analysis using multivariate statistics.

Principal components analysis (PCA) was used initially to assess the overall metabolic differences between the sample groups in an unbiased manner, using the PLS Toolbox (version 5.5.1, Eigenvector Research, Manson, WA, USA) within Matlab (version 7.8; The MathsWorks, Natick, MA, USA). The significance of any group separation along individual principal components was tested using ANOVA and Tukey posthoc tests. Partial least squares discriminant analysis (PLS-DA), a form of supervised multivariate analysis, was performed using the PLS Toolbox, with internal cross-validation (Venetian blinds). The optimal number of latent variables was determined by minimising the classification error and the significance of the predictive models using in-house MatLab scripts. Student t-tests, corrected for a 5% false discovery rate (FDR, Benjamini-Hochberg) using in-house R scripts, were used to confirm the significance of changes in individual mass spectral signals. Corrected p values are reported here as q values.

Signals were putatively annotated with empirical formulae calculated by the MIPack software^69^, searching the KEGG^70^,^71^ and LipidMaps^72^,^73^ databases, and confirmed by performing calculations based on the original spectra in Xcalibur 2.0.7 (Thermo Fisher Scientific). Many signals that contributed significantly to distinguishing the metabolic profiles of individual treatment groups could not be identified by the combination of database and empirical formula searches. Consequently, further identification of selected metabolites was performed using mass spectrometry (same instrument as above). This utilised wide SIM windows and the *m/z* values of identified signals for additional high mass accuracy measurement of unknown signals, narrow SIM (nSIM) windows at up to 4E5 nominal resolution for the determination of isotope patterns, and MS^n^ fragmentation using collision-induced dissociation (CID) and infrared multiphoton dissociation (IRMPD).

### Availability of data and materials

The nontargeted metabolomics data have been deposited at MetaboLights, study number MTBLS91 (ftp://ftp.ebi.ac.uk/pub/databases/metabolights/studies/public/MTBLS91/).

## Additional Information

**How to cite this article**: Mayor, D. J. *et al.* The metabolic response of marine copepods to environmental warming and ocean acidification in the absence of food. *Sci. Rep.* **5**, 13690; doi: 10.1038/srep13690 (2015).

## Supplementary Material

Supplementary Information

Supplementary Table S3

Supplementary Table S6

## Figures and Tables

**Table 1:** Summary of experimental treatments to which 10 replicate groups of 5 *Calanus* spp. were exposed.

time(days)	Temp(°C)	*p*CO_2_(μatm)	pH (NTS) ± SD	TA (μmol/Kg)	Ω_Ar_	Ω_Cal_
t_0_	8	380	8.13 ± 0.00	2734	2.65	4.19
t_0_	10	380	8.11 ± 0.01	2589	2.59	4.07
t_5_	8	380	8.10 ± 0.04–8.09 ± 0.03	2530–2467	2.31–2.21	3.65–3.48
t_5_	8	1000	7.77 ± 0.07–7.76 ± 0.08	2864–2795	1.33–1.27	2.10–2.00
t_5_	10	380	8.09 ± 0.02–8.09 ± 0.04	2459–2459	2.36–2.36	3.71–3.71
t_5_	10	1000	7.78 ± 0.06–7.79 ± 0.10	2910–2982	1.49–1.56	2.34–2.45

Values of total alkalinity (TA), omega aragonite (Ω_Ar_) and omega calcite (Ω_Cal_) were calculated using the average measured salinity across all experimental treatments (34.883 ± SD 0.05) and the reported values of temperature, pCO_2_ and average pH using ‘seacarb’^74^. Hyphenated data represent mean values at the start and end of each 24-hr incubation period. t_0_ = pre-experimental animals; t_5_ = post-experimental animals.

**Table 2:** Summary of the classification error rate and significance of each of the PLS-DA models generated from the FT-ICR mass spectrometric analyses of the polar and nonpolar extracts of *Calanus* spp.

Extracts	Class 1	Class 2	Class error rate (%)	p value
Polar	t_0_, 8 °C	t_0_, 10 °C	52.9	0.500
Polar	t_5_, 8 °C	t_5_, 10 °C	51.1	0.504
Polar	t_5_, 8 °C, 380 μatm *p*CO_2_	t_5_, 8 °C, 1000 μatm *p*CO_2_	59.0	0.683
Polar	t_5_, 10 °C, 380 μatm *p*CO_2_	t_5_, 10 °C, 1000 μatm *p*CO_2_	56.7	0.574
Polar	t_5_, 380 μatm *p*CO_2_	t_5_, 1000 μatm *p*CO_2_	49.9	0.420
Polar	t_0_	t_5_	3.3	p < 0.001
Nonpolar	t_0_, 8 °C	t_0_, 10 °C	35.7	0.141
Nonpolar	t_5_, 8 °C	t_5_, 10 °C	35.1	0.051
Nonpolar	t_5_, 8 °C, 380 μatm *p*CO_2_	t_5_, 8 °C, 1000 μatm *p*CO_2_	51.3	0.66
Nonpolar	t_5_, 10 °C, 380 μatm *p*CO_2_	t_5_, 10 °C, 1000 μatm *p*CO_2_	38.4	0.151
Nonpolar	t_5_, 380 μatm *p*CO_2_	t_5_, 1000 μatm *p*CO_2_	31.7	0.041
Nonpolar	t_0_	t_5_	4.0	p < 0.001

t_0_ = pre-experimental animals; t_5_ = post-experimental animals.
