# Supplementary material for: The metabolic response of marine copepods to environmental warming and ocean acidification in the absence of food
Source: Sci Rep. 2015 Sep 14;5:13690. doi: 10.1038/srep13690 (PMC4650056; doi:10.1038/srep13690)
Supplement: Supplementary Information [file srep13690-s1.doc]

**The metabolic response of marine copepods to environmental warming and ocean acidification in the absence of food**

Daniel J. Mayor^1,4,*^, Ulf Sommer^2^, Kathryn B. Cook^3^, Mark R. Viant^2^

^1^Institute of Biological and Environmental Sciences, Oceanlab, University of Aberdeen, Main Street, Newburgh, Aberdeenshire AB41 6AA, UK;

^2^NERC Biomolecular Analysis Facility – Metabolomics Node (NBAF-B), School of Biosciences, University of Birmingham, Birmingham, B15 2TT, UK.

^3^Marine Scotland Science, Scottish Government, Marine Laboratory, 375 Victoria Road, Aberdeen AB11 9DB, UK.

^4^Ocean Biogeochemistry and Ecosystems, National Oceanography Centre, Southampton, SO14 3ZH, UK.

*Corresponding author. E-mail: [dan.mayor@noc.ac.uk](mailto:dan.mayor@noc.ac.uk)

**Supplementary information**

**A) Statistical analyses of metabolomics data (Table S1-S6; Figs S1 & S2)**

**B) Mass spectrometry based identification of metabolites (Figs S3 - S8; Table S7)**

**C) Mass spectral characterization of further nonpolar compounds (Figs S9 – S11)**

**D) Speculative interpretation of the roles of taurine-containing lipids (Text S1)**

**Supplementary information A: statistical analyses of metabolomics data**

**Table S1.** Principal component analysis for all six classes (polar extracts). Compare Fig. 1 in the main text. The table includes comparisons along the significant principal component PC1 (21.06 % of variance; p-value = 0.018; F-value = 3.018) tested by a Tukey-Kramer post-hoc analysis. There was no significant separation of groups along PCs 2-4. For each principal component individual groups are significantly separated if all 3 values are either positive or negative.

| Group 1 | Group 2 | lower | estimated | upper | significant? |
| --- | --- | --- | --- | --- | --- |
|  |  |  |  |  |  |
| CO2_10C | CO2_8C | -3.9162 | 5.3294 | 14.5750 | No |
| CO2_10C | Ctrl_10C | -5.1303 | 4.1153 | 13.3609 | No |
| CO2_10C | Ctrl_8C | -6.8932 | 2.5925 | 12.0783 | No |
| CO2_10C | t0_10C | -0.3167 | 8.9289 | 18.1744 | Marginal |
| CO2_10C | t0_8C | 0.8873 | 10.1329 | 19.3784 | Significant |
| CO2_8C | Ctrl_10C | -10.2131 | -1.2141 | 7.7849 | No |
| CO2_8C | Ctrl_8C | -11.9825 | -2.7369 | 6.5087 | No |
| CO2_8C | t0_10C | -5.3996 | 3.5994 | 12.5984 | No |
| CO2_8C | t0_8C | -4.1955 | 4.8034 | 13.8024 | No |
| Ctrl_10C | Ctrl_8C | -10.7683 | -1.5228 | 7.7228 | No |
| Ctrl_10C | t0_10C | -4.1854 | 4.8135 | 13.8125 | No |
| Ctrl_10C | t0_8C | -2.9814 | 6.0176 | 15.0165 | No |
| Ctrl_8C | t0_10C | -2.9093 | 6.3363 | 15.5819 | No |
| Ctrl_8C | t0_8C | -1.7053 | 7.5403 | 16.7859 | No |
| t0_10C | t0_8C | -7.7950 | 1.2040 | 10.2030 | No |

**Table S2.** Test results for the PLS-DA model comparing t_0_ versus t_5_ for the polar extracts (see main text, Fig. 2).

**Table S2a:** PLS-DA supervised model (polar extracts), with all 2487 *m/z* values included, built using 6 latent variables and tested using 1000 permutations. These values indicate the validity of the model, highlighting the metabolic differences between the pre-experimental (t_0_) and post-treatment (t_5_) samples.

| Class | Classification error rate (%) | p-value |
| --- | --- | --- |
| t_5_ | 3.3 | <0.001 |
| t_0_ | 3.3 | <0.001 |

**Table S2b**: Optimal  PLS-DA supervised model (polar extracts), comprising of 94 forward selected *m/z* values only, built using 6 latent variables and tested using 1000 permutations. Forward selection maximises the statistical separation of groups by including only the highest ranked variables (*m/z* signals), and permutation testing indicates the validity of the model for this reduced dataset.

| Class | Classification error rate (%) | p-value |
| --- | --- | --- |
| t_5_ | <0.1 | <0.001 |
| t_0_ | <0.1 | <0.001 |

**Table S3:** Signals (*m/z* values) measured in the polar extracts of *Calanus* spp. See separate Excel sheet

**Table S4.** Principal component analysis for all six classes (nonpolar extracts). Compare Fig. 3 in the main text. The table includes comparisons along the significant principal components PC 1 (27.0 % of variance; p-value = 0.004; F-value = 3.991), PC2 (13.7 % of variance; p-value = 0.032; F-value = 2.684), and PC3 (8.0 % of variance; p-value = 0.028; F-value = 2.766) tested by a Tukey-Kramer post-hoc analysis. For each principal component individual groups are significantly separated if all 3 values are either positive or negative.

| Group 1 | Group 2 | lower | estimated | upper | significant? |
| --- | --- | --- | --- | --- | --- |
| PC1 |  |  |  |  |  |
| CO2_10C | CO2_8C | -8.4682 | 4.3758 | 17.2197 | No |
| CO2_10C | Ctrl_10C | -16.7963 | -4.2582 | 8.2799 | No |
| CO2_10C | Ctrl_8C | -8.1550 | 4.6890 | 17.5329 | No |
| CO2_10C | t0_10C | -6.0809 | 6.4573 | 18.9954 | No |
| CO2_10C | t0_8C | -0.0984 | 12.4397 | 24.9778 | Marginal |
| CO2_8C | Ctrl_10C | -20.7789 | -8.6339 | 3.5111 | No |
| CO2_8C | Ctrl_8C | -12.1473 | 0.3132 | 12.7737 | No |
| CO2_8C | t0_10C | -10.0635 | 2.0815 | 14.2265 | No |
| CO2_8C | t0_8C | -4.0810 | 8.0639 | 20.2089 | No |
| Ctrl_10C | Ctrl_8C | -3.1978 | 8.9471 | 21.0921 | No |
| Ctrl_10C | t0_10C | -1.1056 | 10.7154 | 22.5365 | No |
| Ctrl_10C | t0_8C | 4.8768 | 16.6978 | 28.5189 | Significant |
| Ctrl_8C | t0_10C | -10.3767 | 1.7683 | 13.9132 | No |
| Ctrl_8C | t0_8C | -4.3943 | 7.7507 | 19.8957 | No |
| t0_10C | t0_8C | -5.8386 | 5.9824 | 17.8035 | No |
| PC2 |  |  |  |  |  |
| CO2_10C | CO2_8C | -4.9007 | 4.6961 | 14.2930 | No |
| CO2_10C | Ctrl_10C | 0.0900 | 9.4583 | 18.8265 | Significant |
| CO2_10C | Ctrl_8C | -3.3376 | 6.2593 | 15.8561 | No |
| CO2_10C | t0_10C | 0.4647 | 9.8330 | 19.2013 | Significant |
| CO2_10C | t0_8C | -1.0871 | 8.2812 | 17.6494 | No |
| CO2_8C | Ctrl_10C | -4.3124 | 4.7621 | 13.8366 | No |
| CO2_8C | Ctrl_8C | -7.7472 | 1.5631 | 10.8734 | No |
| CO2_8C | t0_10C | -3.9377 | 5.1368 | 14.2114 | No |
| CO2_8C | t0_8C | -5.4895 | 3.5850 | 12.6595 | No |
| Ctrl_10C | Ctrl_8C | -12.2735 | -3.1990 | 5.8755 | No |
| Ctrl_10C | t0_10C | -8.4578 | 0.3747 | 9.2072 | No |
| Ctrl_10C | t0_8C | -10.0096 | -1.1771 | 7.6554 | No |
| Ctrl_8C | t0_10C | -5.5008 | 3.5737 | 12.6483 | No |
| Ctrl_8C | t0_8C | -7.0526 | 2.0219 | 11.0964 | No |
| t0_10C | t0_8C | -10.3843 | -1.5518 | 7.2807 | No |
| PC3 |  |  |  |  |  |
| CO2_10C | CO2_8C | -5.4041 | 1.9386 | 9.2813 | No |
| CO2_10C | Ctrl_10C | -4.6434 | 2.5244 | 9.6923 | No |
| CO2_10C | Ctrl_8C | -5.4701 | 1.8727 | 9.2154 | No |
| CO2_10C | t0_10C | -2.2620 | 4.9059 | 12.0738 | No |
| CO2_10C | t0_8C | 0.6688 | 7.8367 | 15.0046 | Significant |
| CO2_8C | Ctrl_10C | -6.3573 | 0.5858 | 7.5290 | No |
| CO2_8C | Ctrl_8C | -7.1895 | -0.0659 | 7.0576 | No |
| CO2_8C | t0_10C | -3.9759 | 2.9673 | 9.9104 | No |
| CO2_8C | t0_8C | -1.0451 | 5.8981 | 12.8412 | No |
| Ctrl_10C | Ctrl_8C | -7.5949 | -0.6518 | 6.2913 | No |
| Ctrl_10C | t0_10C | -4.3765 | 2.3814 | 9.1394 | No |
| Ctrl_10C | t0_8C | -1.4457 | 5.3122 | 12.0702 | No |
| Ctrl_8C | t0_10C | -3.9099 | 3.0332 | 9.9763 | No |
| Ctrl_8C | t0_8C | -0.9791 | 5.9640 | 12.9071 | No |
| t0_10C | t0_8C | -3.8271 | 2.9308 | 9.6887 | No |

**Table S5.** Test results for the PLS-DA model comparing t_0_ versus t_5_ for nonpolar extracts (see main text, Fig. 4)

**Table S5a:** PLS-DA supervised model (nonpolar extracts), with all 1771 *m/z* values included, built using 5 latent variables and tested using 1000 permutations. These values indicate the validity of the model, highlighting the metabolic differences between the pre-experimental (t_0_) and post-treatment (t_5_) samples.

| Class | Classification error rate (%) | p-value |
| --- | --- | --- |
| t_0_ | 4.0 | <0.001 |
| t_5_ | 4.0 | <0.001 |

**Table S5b**: Optimal  PLS-DA supervised model (polar extracts), comprising of 101 forward selected *m/z* values only, built using 6 latent variables and tested using 1000 permutations. Forward selection maximises the statistical separation of groups by including only the highest ranked variables (*m/z* signals), and permutation testing indicates the validity of the model for this reduced dataset.

| Class | Classification error rate (%) | p-value |
| --- | --- | --- |
| t_0_ | 0.6 | <0.001 |
| t_5_ | 0.6 | <0.001 |

**Table S6:** Signals (*m/z* values) measured in the nonpolar extracts of *Calanus* spp. See separate Excel sheet

**Figure S1:** PLS-DA model of the two groups Ctrl versus CO_2_ for t_5_, for which the 8 °C and 10 °C groups have been combined (nonpolar extracts). The optimal number of LVs was calculated as 4; with a class error rate of 32 %, and a p value of 0.041, this is a marginally significant model.

**
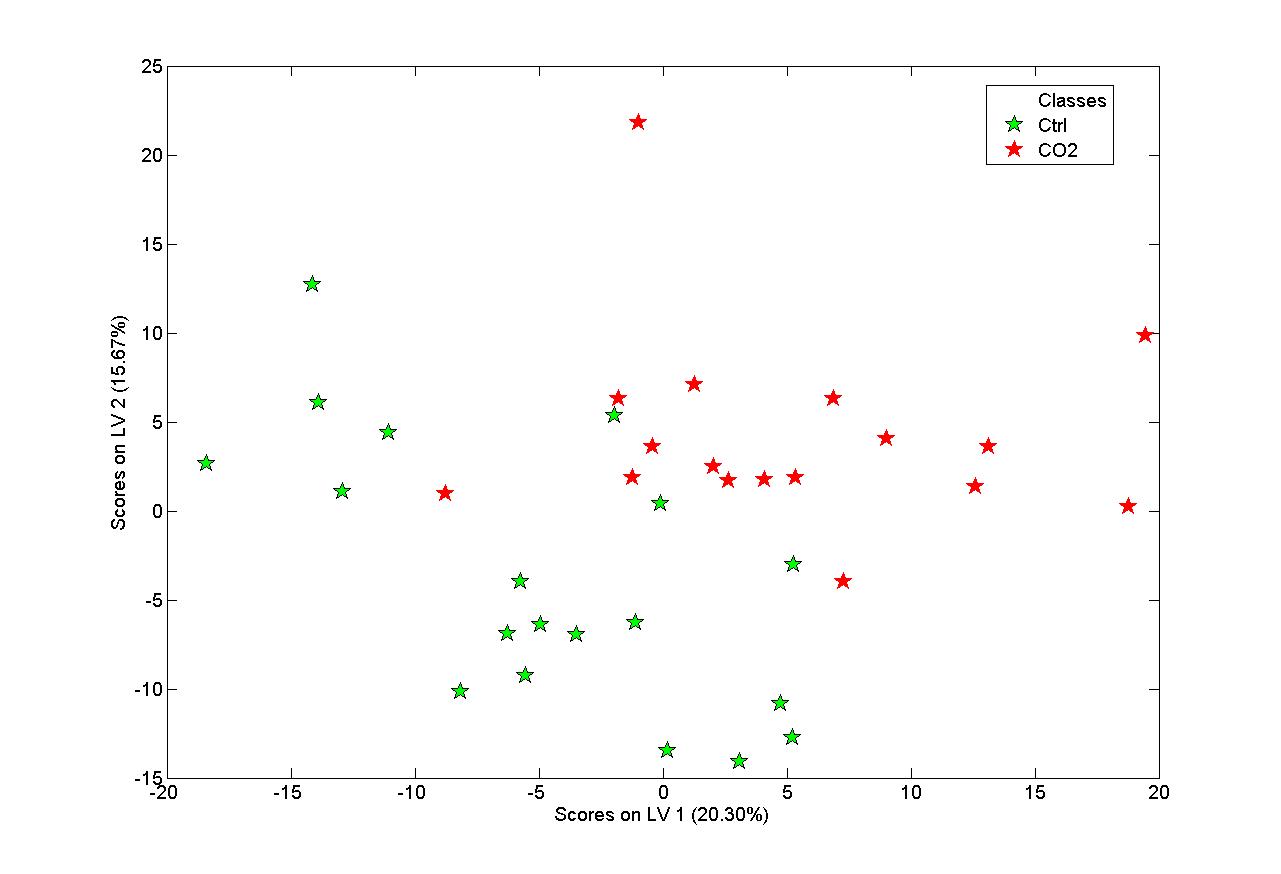
**

**Figure S2:** PLS-DA model of the two groups 8 °C versus 10 °C for which the Ctrl and CO_2_ groups have been combined (nonpolar extracts). The optimal number of LVs was 4; with a class error rate of 35 %, and a p value of 0.051, this is a marginally significant model.
**
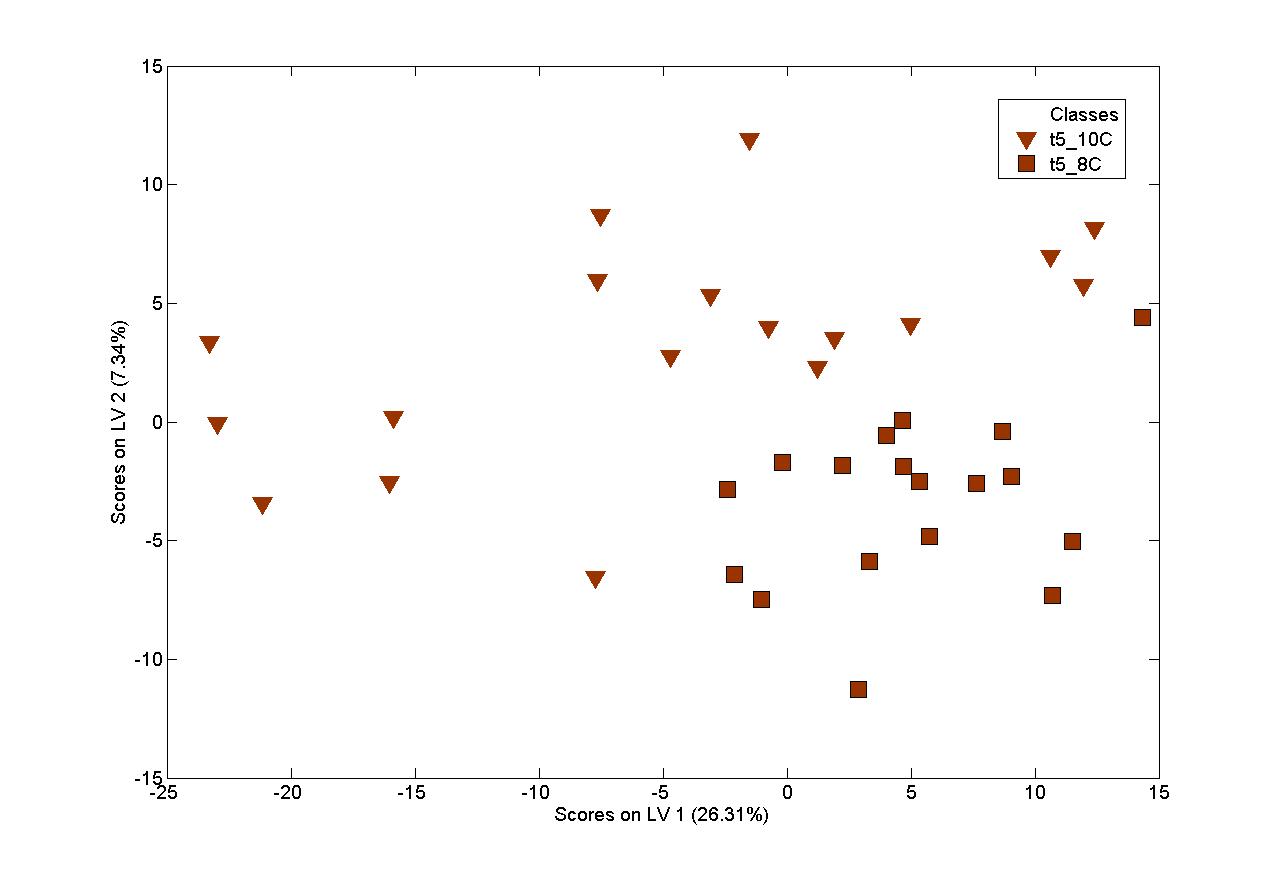
**

**Supplementary information B: Mass spectrometry based identification of metabolites**

**B1) Characterisation / confirmation of polar ions**

**Figure S3.** Confirmation of arginine by CID MS/MS with detection in the ICR cell.

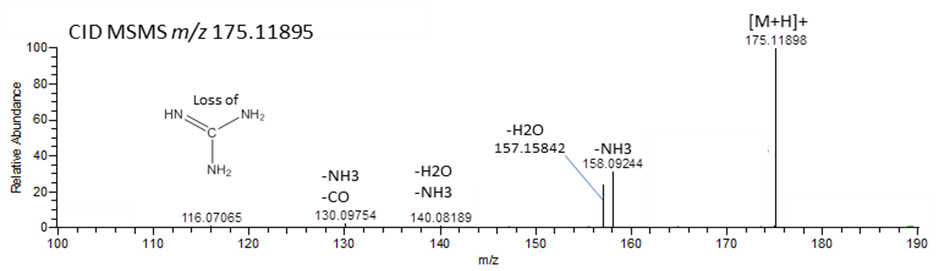


All expected losses from arginine were found.

**Figure S4.** Narrow SIM scan of the ion at *m/z* 288.28731 as part of the characterization of a series of lithiated ions.
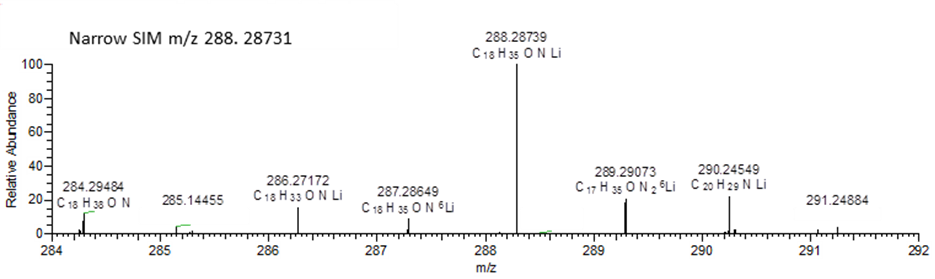


A group of ions from the polar extracts at *m/z* 234.24037, 260.25604, 262.2717, 274.27173, 284.25606, 286.27166, 288.28731 and their isotopic signals could be identified as Li^+^ adducts, as indicated by their ^6^Li isotopes (ca. 8 % intensity at -1.0009 Da). Their composition and relative intensities match those of a highly abundant fatty acid amide series in the spectra, which had been removed by the blank filter, and they are therefore likely not of biological interest.

**B2) Characterization of the taurine lipid structures**

Among the significantly changing signals were some that clearly belonged to a series of closely related compounds, with the C_2_H_4_, H_2_, C_2_H_2_ etc. distances typical for a lipid series, but which did not give any acceptable matches with existing databases.

**Table S7.** List of taurine lipids (^13^C isotopes not listed; see Table S6). Columns: q value – t-test p value after FDR correction; fold.change: time point t_5_ by time point t_0_; VarImp: variable importance PLS-DA model. C14:0 etc. in the annotation column are based on the corresponding fatty acid side chains in the molecule.

**MS identification work:**
2.A) Narrow selected ion monitoring (SIM) by FT-ICR mass spectrometry

**Figure S5.** Narrow SIM window around the ion at *m/z* 686.50340 at nominal 200k resolution


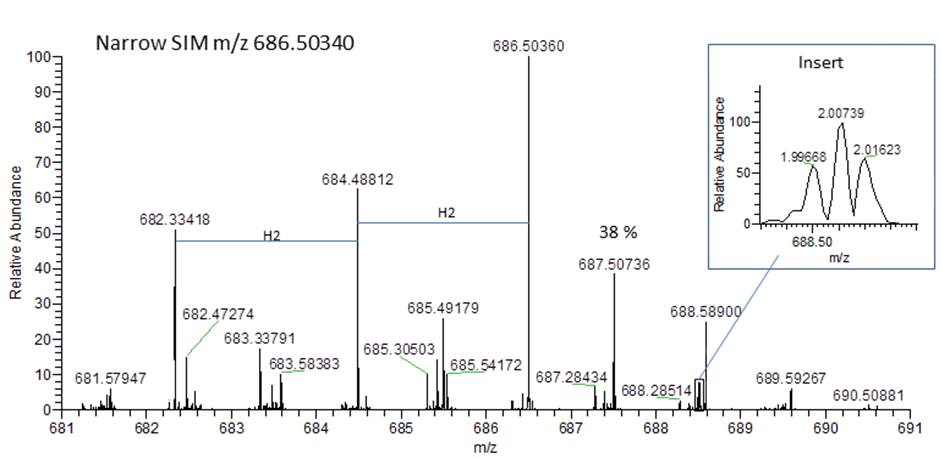


The MS spectrum shows the lipids appearing in a typical lipid pattern of signals at a distance of two protons. Concentrating on the main signal at ion at *m/z* 686.50340, at 2E5 nominal resolution (at *m/z* 400), an isotopic signal at 1.997 Da could be resolved. While ^34^S should have been at 1.996 Da, the ^13^C_2_ isotope and the +2H ion are shifted in a similar way. The intensity of 4 % of the monioisotopic signal speaks for the presence of one sulfur atom in the molecule.

2.B) MS fragmentation (MS/MS) experiments, using Infrared Multiphoton Dissociation (IRMPD):

**Figure S6.** IRMPD MS/MS of the ions at *m/z* 686, 684, and 732


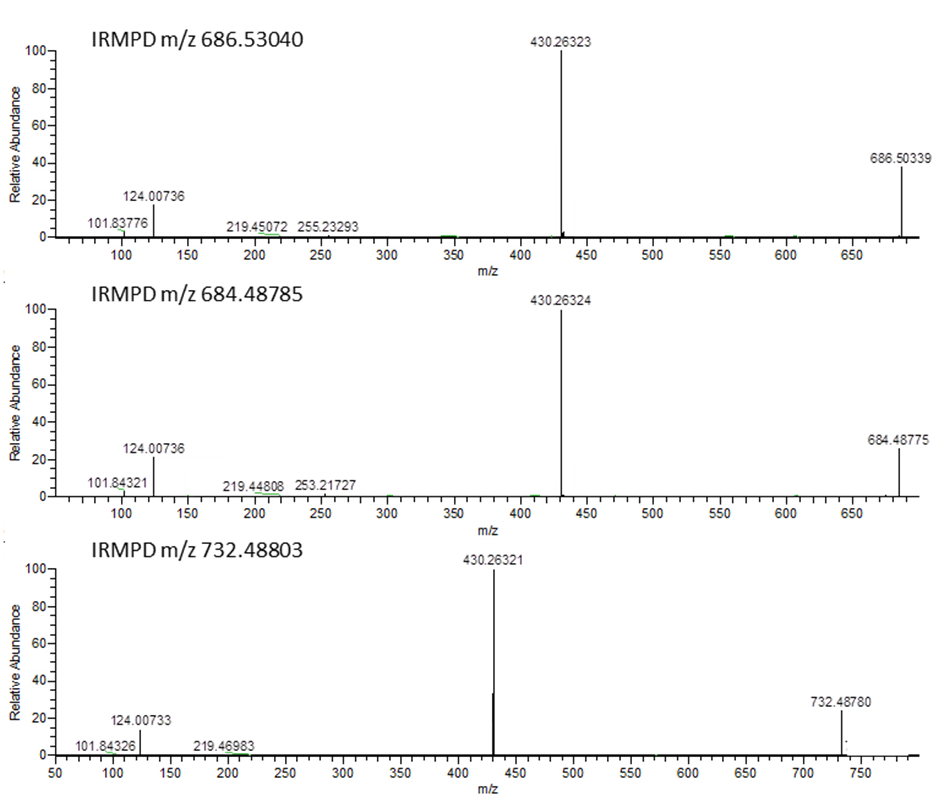


All fragmented ions showed a primary fragment at *m/z* 430.26323 after a neutral loss corresponding to a fatty acid as indicated in the table above. The small signals at 255.23293 and 253.21727 match these fatty acid losses corresponding to palmitic and palmitoleic acids. The second fragment ion corresponds to the mass of taurine (124.00739 Da). The signals around 101 and 219 are known electronic noise.

2.C) MS fragmentation (MS/MS and MS^n^) experiments, using Collision-Induced Dissociation (CID):

**Figure S7.** CID MS/MS to MS^4^ fragmentation. While the MS/MS fragments were detected in the ICR cell, the further stages were detected b the LTQ ion trap detector.


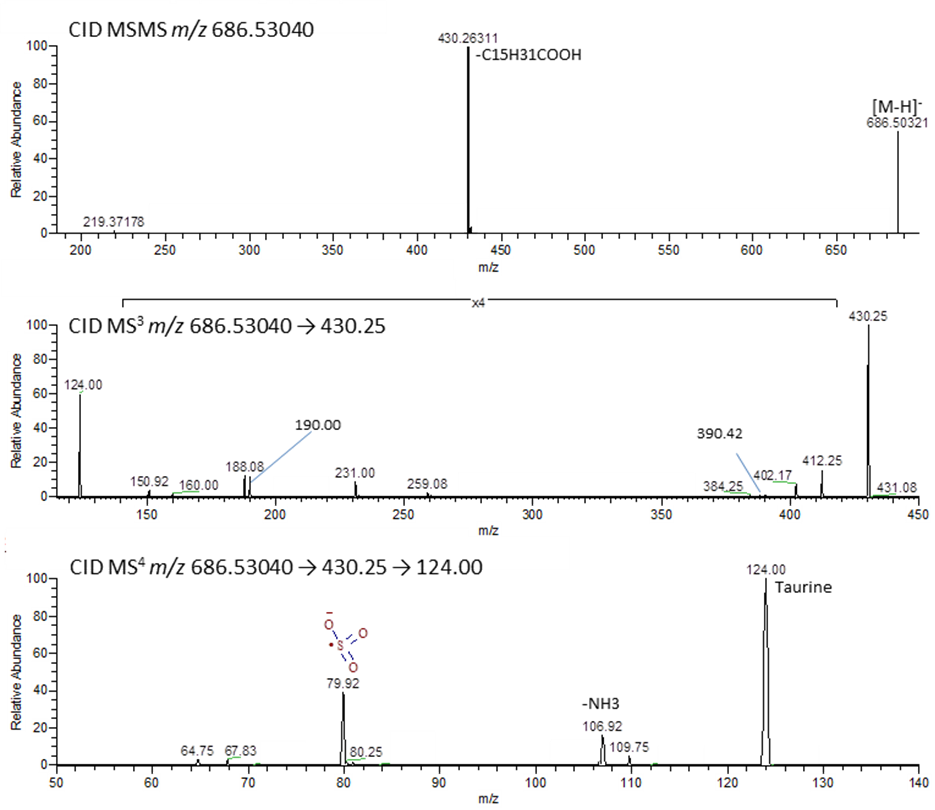


The MSMS spectrum with ICR detection is expectedly very similar to the IRMPD spectrum, with the lower third of the m/z range cut off. The MS^3^ spectrum here shows that the *m/z* 124 ion, which corresponds to the mass of taurine (124.00739), is derived from the *m/z* 430 ion. The MS^4^ spectrum contains the fragments that would be expected from taurine (Metlin database, see below). Thanks to the high resolution and mass accuracy in the IRMPD data, the compositions of these ions can be calculated with good reliability - the 430 Da fragment is likely [C22H40NO5S]^-^ (430.26327 Da). Subtraction of taurine ([C2H6NO3S]^-^) leaves an unknown part of about 306 Da (306.25588, [C20H34O2]), which is also the best calculated mass. The low intensity signals are one-spike noise. No further ions were found in the MS spectra that corresponded related directly to the deacylated *m/z* 430 ion, but an ion at *m/z* 448.27436 corresponding to the lysolipid.
While the variable part corresponds to common fatty acids and the *m/z* 124 fragment to taurine, the data does not provide much information about the 306 Da structure beyond its fairly simple composition. Fragmentation shows losses of water (-18), likely elimination of the OH group, and of CO or C_2_H_4_ (-28) which can be followed by a water loss. According to the decimals in the ion trap data, this initial loss of 28 Da would rather be expected as loss of a C_2_H_4_ group.
The closest fragmentation spectra to these expected ones are N-acyl taurines in the Metlin database (http://metlin.scripps.edu/metabo_advanced.php), all of which show the fragments for taurine (m/z 124.007, 106.981, and 79.957), and the nonsaturated species also *m/z* 150.994 (C3H5NO4S^-^). The two ions detected at m/z 188.08 and 190.00 still contain nitrogen, with the latter possibly having lost the sulfonate group. The interpretation of the rest of the fragments will be ambiguous, with still an array of different structures and fragmentation mechanisms possible.
Bile acids like taurocholic acid are too large to fit this structure, but there are open-chain taurolipids described in the protozoan *Tetrahymena thermophila* that would approximately match the measured size and composition. The proposed biosynthesis starts with stearic acid and taurine, and sequential formation of double bonds and their hydroxylation leads via lipotaurine to lysotaurolipids A to C^(1)^. Figure SI12 below shows the structure of Taurolipid A.

**Figure S8a.** Taurolipid A from *T. thermophila5*.

After elimination of the palmitic acid side chain in the figure, the remaining structure (lipotaurine) would have the composition C20H38NO6S(-), which by moderate modification could be transformed into a molecule of the [C22H40NO5S]^-^ composition we found for the copepod taurine lipids.
Other mostly linear structures have been described in crab2 and in several kinds of bacteria (listed in ref. 3), but are too complex in order to resemble the copepod structure.

Purification and identification by NMR analysis of these relatively abundant taurine lipids would have been possible, but as we were aware that collaborators in other research groups were close to solving the structure of these lipids4, we decided not to pursue their further analysis ourselves. Among the eight lipids listed in this paper, our findings match half, those with a double bond at R^1^; the others are likely at too low a concentration to be robust enough to pass our peak filtering. We detected additional species due to the different treatments analysed.


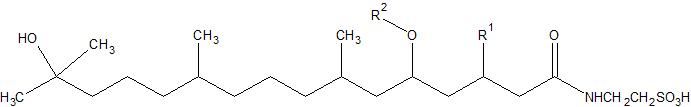


**Figure S8b.** General copepodamide structure (after ref. 4).

**Supplementary information C: Mass spectral characterization of further nonpolar compounds**

Confirmation of phosphatidylethanolamines (PE lipids):

**Figure S9.** Confirmation PE 38:6 by IRMPD MS/MS. PE lipids are isomeric to phosphatidylcholines (PC lipids) of shorter chain lengths. This species contains almost exclusively palmitic acid and DHA residues, and is therefore clearly a PE lipid. The signal at m/z 191 is present in the Metlin database (http://metlin.scripps.edu), but not annotated.


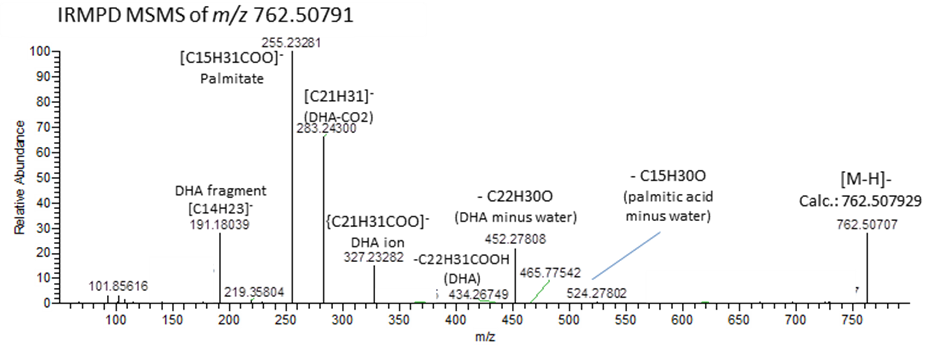


Confirmation of phosphatidylcholines (PC lipids):

**Figure S10.** Confirmation of PC lipid PC 36:5 by CID MS/MS and MS^3^ spectra. Acetate adducts of PC lipids are isomeric to deprotonated phosphatidylserines (PS lipids). The high-resolution MS/MS spectrum shows the combined losses of acetic acid and methane that are expected from PC lipid acetate adducts. The lower resolution MS3 spectrum shows an almost exclusive composition of palmitic acid and EPA. The lower abundant ions in the MS/MS spectrum are electronic noise.
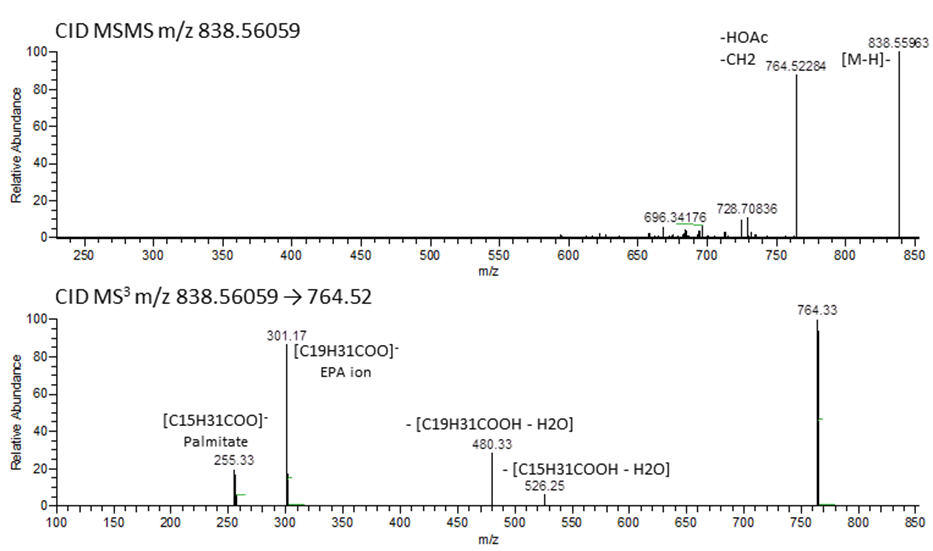


Confirmation of free fatty acids:

**Figure S11.** Confirmation of DHA by CID MS/MS and MS^3^ spectra. Free fatty acids and fatty acid alkyl esters are isomeric. Fragmentation of the *m/z* 327 ion leads to the expected losses of water and CO_2_. Further fragmentation of the resulting in a pattern consistent with polyunsaturated hydrocarbon chains; no authentic standard was run. The m/z 191 and 115 ions are also present in Figure S9 (the latter not annotated because of low intensity).


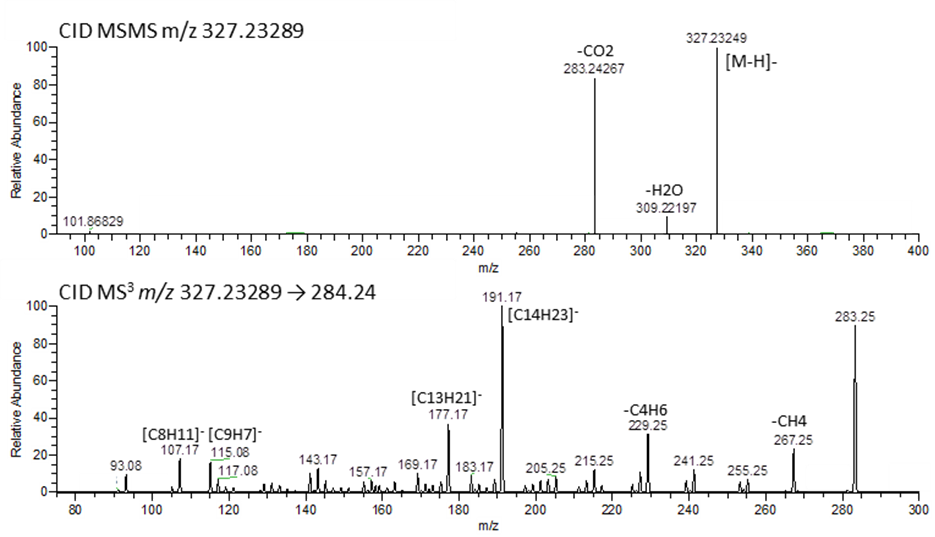


**Supplementary information C: References**

1. Kaya, K. & Sano, T. Definition of total biosynthesis pathway of taurolipids in *Tetraheymena* cells. *Biochim. Biophys. Acta*, 1084, 101-104 (1991).

2. Van der Oord, A., Danielsson, H. & Ryhage, R. On the structure of the emulsifiers in gastric juice from the crab, *Cancer pagurus L..* *J. Biol. Chem.,* 245 (5), 2242-7 (1965).

3. Denger, K., Mayer, J., Hollemeyer, K. & Cook, A. M. Amphoteric surfactant N -oleoyl-N -methyltaurine utilized by *Pseudomonas alcaligenes* with excretion of N –methyltaurine. *FEMS Microbiol. Lett.,* **288**, 112-117 (2008).

4. Selander, E. et al. Predator lipids induce paralytic shellfish toxins in bloom-forming algae. *Proc. Nat. Acad. Sci*., 112, 6395-6400 (2015).

5. Kaya, K. Chemistry and biochemistry of taurolipids. *Prog. Lipid Res.*, 31, 87-108 (1992).

Supplementary information D: Speculative interpretation of the roles of taurine-containing lipids in *Calanus* spp.

**Text S1.** Our speculative interpretation of the role of the taurine-containing lipids is based around the apparent anti-oxidative effects of taurine and the role that taurine-bearing compounds are known to play in the digestion and absorption of fats1. Taurine conjugates with a range of organic acids to form compounds that have detergent-like properties, including mammalian bile acids and the acylsarcosyltaurines found in the gastic juice of crabs2^,3^ (see Ref. 4 for the structures of a range of other naturally-occurring taurolipids). All moieties of fatty acids associated with the taurine-containing lipids, apart from the ω-3 polyunsaturated fatty acids (PUFAs) eicosapentaenoic acid (EPA, 20:5(ω-3)) and docosahexaenoic acid (DHA, 22:6(ω-3)) (denoted 20:5 and 22:6 respectively herein), increased significantly over the duration of our experiment. This suggests that taurine-containing lipids play an active role in the transportation and protection of metabolic substrates from their storage location to the point of oxidative catabolism within mitochondria. A similar role for taurine-conjugated fatty acids has been hypothesized for the transmembrane transport of these metabolites in bacteria4. It is interesting to note that while many studies make reference to the fact that calanoid copepods sequester enormous quantities of fatty acids as wax esters in a central lipid sac, few consider how these metabolic reserves are transported to and from their storage location. The molecular formula of the C_20_H_34_O_2_ neutral loss is consistent with dihomo-γ-linolenic acid (DGLA, 20:3(ω-6)), a fatty acid that is known to yield anti-inflammatory eicosanoids6 and interfere with the production of other eicosanoids that have inflammatory effects7. It also has wider effects on lipid metabolism in humans, the effects of which are mediated by EPA and γ-linolenic acid (GLA, 18:3(ω-6)) ^(Refs 8, 9)^. It seems at least plausible that an organism up-regulating fatty acid metabolism owing to starvation may attempt to offset the increased oxidative stress associated with lipid peroxidation by coupling the delivery of catabolic substrates with the supply of anti-inflammatory DGLA6 and anti-oxidative taurine1. Partial support for this hypothesis comes from the observation that taurine can prevent oxidative stress due to lipid peroxidation10, potentially by stabilising the oxidative environment within mitochondria and thus preventing the leakage of reactive compounds11.

**Supplementary information D: References**

1. Huxtable, R. J. Physiological actions of taurine. *Physiol. Rev.*, **72**, 101-163 (1992).

2. Vonk, H. J. Emulgators in the digestive fluids of invertebrates. *Arch. Int. Physiol. Bio.* **70**, 67-85 (1962).

3. Van der Oord, A., Danielsson, H. & Ryhage, R. On the structure of the emulsifiers in gastric juice from the crab, *Cancer pagurus L..* *J. Biol. Chem.,* **245** (5), 2242-7 (1965).

4. Denger, K., Mayer, J., Hollemeyer, K. & Cook, A. M. Amphoteric surfactant N -oleoyl-N -methyltaurine utilized by *Pseudomonas alcaligenes* with excretion of N –methyltaurine. *FEMS Microbiol. Lett.,* **288**, 112-117 (2008).

5. Kaya, K. Chemistry and biochemistry of taurolipids. *Prog. Lipid Res.*, 31, 87-108 (1992).

6. Fan. Y. Y & Chapkins, R. S. Importance of dietary gamma-linolenic acid in human health and nutrition. *J. Nutr.*, **128**, 1411-1414 (1998).

7. Belch, J. J. F. & Hill, A. Evening primrose oil and borage oil in rheumatologic conditions. *The American Journal of Clinical Nutrition*, **71,** 352S-356S (2000).

8. Barham, J. B., Edens, M. B., Fonteh, A. N., Johnson, M. M., Easter, L. & Chilton, F. H. Addition of eicosapentaenoic acid to gamma-linolenic acid-supplemented diets presents serum arachidonic acid accumulation in humans. *J. Nutr.*, **130**, 1925-1931 (2000).

9. Johnson, M. M. et al. Dietary supplementation with gamma-linolenic acid alters fatty acid content and eicosanoids production in healthy humans. *J. Nutr.*, **127**, 1435-1444 (1997).

10. Zhang, M. et al. Role of taurine supplementation to prevent excercise-induced oxidative stress in healthy young men. *Amino acids*, **26**, 203-207 (2004).

11. Hansen, S. H., Andersen, M. L., Birkedal, H., Cornett, C. & Wibrand, F. The important role of taurine in oxidative metabolism. *Adv. Exp. Med. Biol.*, **583**, 129-135 (2006).
